# Comparing Real and Virtual Nature Exposure on Cognition, Well-Being, and Brain Activity in Adults With and Without Attention-Deficit/Hyperactivity Disorder: Protocol for a Randomized Experimental Study

**DOI:** 10.2196/82970

**Published:** 2026-05-22

**Authors:** Tianran Zhang, Dimitrios Adamis, Natasha Langan, Martin O’Neill

**Affiliations:** 1 Atlantic Technological University Department of Health and Nutritional Sciences Sligo Ireland; 2 Atlantic Technological University The Health and Biomedical Strategic Research Centre (HEAL) Sligo Ireland; 3 HSE Sligo Leitrim Mental Health Services Sligo Ireland

**Keywords:** ADHD, attention-deficit/hyperactivity disorder, cognition, EEG, electroencephalography, nature exposure, neuroscience, psychology, virtual reality, VR

## Abstract

**Background:**

Natural environments are associated with improved cognitive functioning and psychological well-being, potentially through attentional restoration and stress reduction. Virtual reality (VR) offers an accessible way to simulate natural settings; however, it remains unclear whether VR nature engages the brain and cognition in the same way as real nature, particularly in adults with attention-deficit/hyperactivity disorder (ADHD), who show atypical neural oscillations and heightened sensitivity to environmental demands.

**Objective:**

This protocol describes a randomized experimental study designed to compare the effects of a single exposure to real vs virtual nature on cognition, well-being, and brain activity in adults with and without ADHD and to examine how neurocognitive profiles and ADHD symptom dimensions shape these responses over time.

**Methods:**

A total of 80 adults (40 with a confirmed ADHD diagnosis and 40 neurotypical controls) will be recruited and randomly assigned to either a real nature condition or an immersive VR-simulated nature condition. After a baseline assessment, participants will complete a 20-minute seated exposure in the assigned condition while 32-channel mobile electroencephalography (EEG) is recorded. Immediately post exposure, they will complete gamified cognitive tasks assessing cognitive flexibility and metacognition, self-report measures of mood, perceived restoration, simulator sickness, and nature connectedness. ADHD symptomatology and functional impact will be assessed with standardized scales at preexposure and postexposure and at the final follow-up. Ecological momentary assessment of emotional well-being, weekly self-reports of real-world nature and VR exposure, and repeated nature connectedness ratings will be collected via a mobile app over an 8-week follow-up period to characterize the decay or maintenance of effects. EEG analyses will focus on alpha-band markers of cognitive restoration, complemented by dimensional modeling of ADHD symptoms in linear mixed-effects models. The study has been preregistered on the Open Science Framework and will be prospectively registered with the Australian New Zealand Clinical Trials Registry (ANZCTR) before enrollment of the first participant. Ethical approval has been received from the Research Ethics Committee at Sligo University Hospital.

**Results:**

At the time of manuscript submission, participant recruitment had not yet begun and no outcome data were available. The project received funding in May 2025. Participant recruitment is expected to begin in January 2026, followed by data collection in April 2026 to ensure suitable outdoor conditions. Data analysis will follow data collection, and study findings are expected to be published in May 2028.

**Conclusions:**

This study will test whether VR nature can reproduce key cognitive and neural benefits of real-world nature exposure and whether these effects differ between adults with ADHD and neurotypical adults. By integrating EEG, cognitive performance, symptom dimensions, and longitudinal self-report data, the findings are expected to clarify when VR nature may serve as a useful, scalable complement to real nature–based approaches for supporting attention and psychological well-being.

**International Registered Report Identifier (IRRID):**

PRR1-10.2196/82970

## Introduction

Humans evolved in close connection with the natural world, and growing evidence suggests that contact with nature continues to play an important role in supporting psychological well-being. As mental health challenges increasingly emerge within the built structures of modern cities, many have turned their attention back to nature in search of restoration. A substantial body of research has shown that exposure to natural environments is associated with improvements across multiple domains, including cognitive and brain functioning, autonomic regulation, sleep quality, and reductions in stress, anxiety, depression, anger, and fatigue [1-8]. These benefits are often apparent in the cognitive domain: compared with urban settings, natural environments have been linked to enhanced attention, executive functioning, and perceived restorativeness [9-14]. Recent meta-analytic findings further indicate that even brief exposures as short as 10 minutes can produce measurable short-term benefits, especially for adults with mental health conditions [1,15]. Moreover, the presence of green or natural spaces in one’s surroundings has been linked to quantifiable psychological benefits [16].

These observations align with longstanding theoretical perspectives suggesting that humans may have an inherent affinity for nature. The biophilia hypothesis proposes that, across evolutionary history, natural settings supported survival and thus shaped a preference for the sensory qualities of natural landscapes. Building on this foundation, 2 major psychological frameworks have guided contemporary research on the mental effects of nature. Attention Restoration Theory (ART) describes how natural environments may replenish directed attention by engaging effortless, involuntary forms of attention, often referred to as “soft fascination” [17]. Early experimental work consistent with ART showed that time spent in natural settings was followed by improved performance on demanding cognitive tasks [9]; although this evidence was primarily behavioral, it explicitly linked the effects to prefrontal functioning and helped pave the way for subsequent functional magnetic resonance imaging (fMRI) and electroencephalography (EEG) studies of nature exposure. Stress Reduction Theory (SRT), developed around the same period, is grounded in psychoevolutionary and psychophysiological research and emphasizes rapid, precognitive affective responses to natural stimuli [18]. Classic studies comparing responses to natural vs urban scenes reported lower autonomic arousal indexed by measures such as heart rate, skin conductance, and facial muscle activity, supporting the idea that such settings can quickly dampen stress responses through parasympathetic activation [19].

Building on these theoretical accounts, recent work has begun to test whether exposure to natural environments is accompanied by the kind of bottom-up, low-effort processing proposed by ART and SRT. Neuroimaging studies have reported changes in prefrontal activity when people spend time in, or view, natural settings compared with urban ones [16,20-22]. Mobile EEG studies conducted during outdoor walks further suggest that moving from busy city streets into green spaces is followed by a rapid decrease in neural indices associated with frustration and arousal and a simultaneous increase in alpha activity linked to more relaxed, meditative states [23]. This pattern is consistent with the idea that responses can shift automatically in natural environments, as described by SRT, while involuntary attention takes over from effortful control, as described by ART.

Although the neural pathways through which real natural environments influence cognition are not fully understood, advancements in immersive technology have made it possible to study simulated nature using VR. Motivated by the proven benefits of nature exposure, as well as the unequal accessibility of natural spaces, researchers have increasingly turned to VR as a scalable, affordable, and portable alternative for understanding neuroplasticity [24] and promoting public well-being [25]. VR simulations of forests, coasts, and other natural settings can reduce stress and anxiety, support relaxation and emotional regulation, and even alleviate pain [26-29]. In the cognitive domain, VR-simulated nature has been associated with higher perceived restorativeness and improvements in working memory and executive function [26,30-32]. Even minimal natural cues, such as the inclusion of virtual plants, have been linked to improved cognitive performance and emotional well-being [33]. As head-mounted displays have become more immersive and embodied, VR nature paradigms are increasingly combined with neurophysiological tools such as EEG to characterize cognitive engagement, attentional load, emotional processing, and other mental states, with applications ranging from clinical interventions to neurorehabilitation [34-36].

Despite promising findings, whether VR-based nature engages the same processes as real nature remains unclear. VR nature experiences often lead to self-reported relaxation, but physiological data do not always show a simple parallel. One study reported that VR forest scenes led to greater improvements in mood than VR urban scenes, while reductions in blood pressure and heart rate were similar across conditions, suggesting that subjective restoration was not fully mirrored in basic physiological responses [37]. An EEG study found that watching forest videos in VR produced changes in relative alpha and beta power, particularly an increase in the ratio of sensorimotor rhythm-mid beta to theta, which the investigators interpreted as reflecting concentration and immersion in the VR content rather than simple relaxation [38]. These findings suggest that VR nature may rely more on an actively engaged, top-down route to feeling restored rather than the largely effortless attentional shift described by ART. Another EEG study on top-down processing and nature connectedness provides a complementary perspective. It showed that when participants heard the same ambiguous environmental sound, simply being told it came from a “natural waterfall” vs an “industry” led to different alpha responses and feelings of relaxation [39]. This finding indicated that beliefs and cognitive labels about nature can modulate physiological reactions even when the sensory input is identical. Together, these results raise the possibility that VR-simulated nature often achieves subjective restoration through a more cognitively engaged, top-down route, whereas real nature may more readily trigger bottom-up, low-effort recovery consistent with these theories. To date, however, there has been no direct EEG comparison of real and VR or other simulated nature environments. One related near-infrared spectroscopy study found that viewing real plants, but not projected images of the same plants, produced a marked reduction in prefrontal oxyhemoglobin concentration [40], suggesting that genuine, physically present nature may more easily induce prefrontal deactivation than visually similar simulations.

This hypothesis is supported by broader EEG work in immersive VR, which suggests that the medium of VR itself can change how the brain handles sensory and cognitive demands, but the direction of these effects is not consistent. Conceptual studies have argued that, although VR can engage brain systems similar to those involved in real-world interaction, maintaining a convincing sense of presence requires ongoing monitoring and correction by prefrontal networks [34]. Reviews of EEG in immersive VR similarly note that situations with sensory conflict or rich interactivity are often accompanied by increased frontal and central theta and reduced alpha, patterns typically linked to sustained attention and task engagement rather than passive rest [35,41]. Some studies report that the sensory richness of immersive VR may impose higher cognitive load and, in specific tasks, impair performance or alter neural processing, for example, reduced spatial selectivity of hippocampal place cells in 2D VR, higher mental workload in immersive laparoscopic surgery training, or lower learning outcomes at very high levels of presence [42-44]. Conversely, other studies indicate that immersive VR can reduce cognitive load or show no disadvantage compared with traditional digital formats, with users reporting higher motivation and usability [45-47]. The inconsistent evidence suggests that immersive VR is not a neutral substitute for real-world experience. It can be engaging and potentially relaxing, but its cognitive and neural consequences depend strongly on context, and it remains unclear whether it supports the same low-effort restorative processes proposed for real nature exposure.

Although the mechanisms by which different environments influence cognition are not yet fully understood, VR has already been widely used to assess and treat diverse neurocognitive profiles, particularly attention-deficit/hyperactivity disorder (ADHD), for example, through virtual classroom paradigms and aquatic attention tasks [48,49]. ADHD is a neurodevelopmental condition marked by atypical neural oscillations, altered prefrontal activation, and persistent difficulties in sustained attention and executive function [50]. Individuals with ADHD are sensitive to environmental distractions and cognitive load [51-53], so high stimulation in immersive VR might be expected to be problematic. Yet clinical reports indicate that many people with ADHD tolerate and even prefer VR environments, and VR-based motor-cognitive and classroom-like programs have been shown to be feasible and associated with improvements in selected behavioral and cognitive outcomes [54]. Consistent with this, a recent meta-analysis concluded that brief (15-25 minutes) VR assessments or longer interventions are generally safe and acceptable for children with ADHD, while highlighting the need for long-term and mechanistic studies [55]. One possible explanation comes from optimal stimulation and arousal-dysregulation accounts of ADHD, which propose that symptoms partly reflect chronically low or unstable arousal and that richer, more engaging stimulation can temporarily normalize performance [56,57]. Consistent with this view, several studies report that when tasks are delivered with higher motivational salience or immersive, game-like formats, including VR-based interventions, children with ADHD show improved attention and executive performance compared with lower-stimulation or nonimmersive formats [58,59]. VR may therefore enhance short-term engagement in ADHD, for example by providing salient cues and reducing external distractions, but these benefits do not imply that immersive VR is intrinsically restorative or low in cognitive demand, and the underlying mechanisms likely depend on specific task demands and sensory load. Meanwhile, although no studies to date have directly examined VR nature exposure in ADHD, emerging evidence suggests that contact with real natural environments may improve attention and emotional regulation in this population [60].

This view aligns with dimensional frameworks such as the Research Domain Criteria [61], which emphasize the distributed neurobiological underpinnings of attentional and regulatory processes across clinical and nonclinical populations, and dual-pathway models that highlight heterogeneous cognitive and motivational routes to ADHD symptoms [62]. Studying ADHD-related traits along continuous spectra, rather than only using categorical diagnoses, allows us to examine whether variation in symptom levels is linked to variation in environmental sensitivity and restorative responses. From an ecological standpoint, comparing adults with ADHD and neurotypical controls in both real nature and VR nature settings provides a practical way to test how attentional and affective mechanisms operate in real-world-like environments. Natural settings may preferentially engage bottom-up sensory processing and low-effort neural restoration, whereas VR nature may rely more on cognitively engaged, top-down routes. By modeling ADHD symptom dimensions across the full sample and including diagnostic group, this study aims to characterize where responses to real and virtual nature converge and where they diverge and to identify conditions under which VR-based nature exposure is more or less suitable for individuals with higher ADHD traits.

The overarching aim of this study is to test whether immersive VR-simulated nature can approximate the cognitive and neural benefits of real-world nature and to identify for whom each modality is most suitable. To this end, we focus on 3 core questions. First, we ask whether a brief exposure to real and VR-simulated nature produces comparable immediate changes in attention, cognitive flexibility, affect, and EEG markers of restoration relative to baseline or control conditions. Second, we examine whether adults with ADHD differ from neurotypical adults in these cognitive, affective, and neural responses to real and virtual nature. Third, we investigate, across the full sample, how continuous ADHD symptom dimensions and related traits predict the magnitude and durability of benefits from each type of nature exposure.

Based on the literature and the theoretical framework outlined above, we hypothesize that both real and VR-simulated nature will acutely improve attention, cognitive flexibility, and mood and will be accompanied by EEG changes consistent with restoration, but that real nature will show stronger signatures of low-effort, bottom-up restoration than VR. We further expect that adults with ADHD will show equal or greater cognitive and affective gains than neurotypical adults after both real and VR-simulated nature, while displaying distinct patterns of prefrontal and parietal EEG change, reflecting different regulatory mechanisms. More tentatively, we anticipate that individual differences in ADHD-related symptoms will shape both the size and the time course of these effects: higher symptom levels may be linked to larger immediate benefits from nature exposure, but also to a faster decline of gains over the follow-up period, and the pattern of attenuation may differ between real and virtual nature along the continuum from neurotypical adults to those with more pronounced ADHD traits. Together, these aims position the study to clarify where responses to real and virtual nature converge, where they diverge, and under which conditions VR-based nature exposure is a suitable supplement to real-world nature for adults with higher ADHD traits.

## Methods

### Ethical Considerations

The study was preregistered on the Open Science Framework [63] on June 11, 2025, with the protocol and planned analyses made publicly available before data collection. The study will also be prospectively registered with the Australian New Zealand Clinical Trials Registry (ANZCTR) before enrollment of the first participant. Ethical approval was obtained from the Research Ethics Committee at Sligo University Hospital on April 7, 2025 (reference number 1044). Any substantial protocol amendments (eg, changes to eligibility criteria, assessments, or exposure procedures) will be submitted for prior review and approval by the same committee and will be updated in the registrations before implementation. Given the low-risk, nontherapeutic nature of this trial, no special ancillary or posttrial care is planned beyond usual clinical care; however, participants who experience distress will be advised to contact their treating clinicians and will be provided with information on appropriate local support services. Protocol version 1.4 dated January 2026.

### Study Design

This study uses a randomized experimental design with a between-subjects factor and a within-subject repeated-measures component over an 8-week follow-up period. Participants will be randomly assigned to 1 of 2 exposure conditions: a real natural environment or an immersive VR-simulated natural environment. Cognitive performance, emotional well-being, nature connectedness, and ADHD symptomatology will be assessed both before and immediately after exposure, with additional follow-up assessments conducted over the next 8 weeks, while brain activity will be continuously recorded during the exposure session.

### Procedures

Participant enrollment will be conducted through a face-to-face clinical interview with a trained psychologist or psychiatrist to confirm the diagnosis of ADHD and assess potential comorbidities based on *DSM-5* (*Diagnostic and Statistical Manual of Mental Disorders, Fifth Edition*) criteria.

Following informed consent and completion of baseline assessments, participants will be stratified by diagnostic status (ADHD vs neurotypical control). Within each stratum, the random allocation sequence will be generated by an independent research coordinator (not involved in recruitment or outcome assessment) using computer-generated permuted blocks of variable size with a 1:1 allocation ratio. This stratified block design ensures balanced group sizes over time and equal distribution of diagnostic groups across conditions.

The allocation sequence will be implemented using sequentially numbered, opaque, sealed envelopes prepared in advance by the independent research coordinator. Treating clinicians or research assistants will enroll participants and open the next envelope only after baseline measures are completed, ensuring that upcoming assignments remain concealed from both participants and enrollment staff. Due to the nature of the intervention condition, blinding of participants and experimenters is not feasible; however, data analysts will remain blinded to group allocation and condition labels until the primary analyses are completed to minimize bias during statistical analysis.

**Figure 1:**
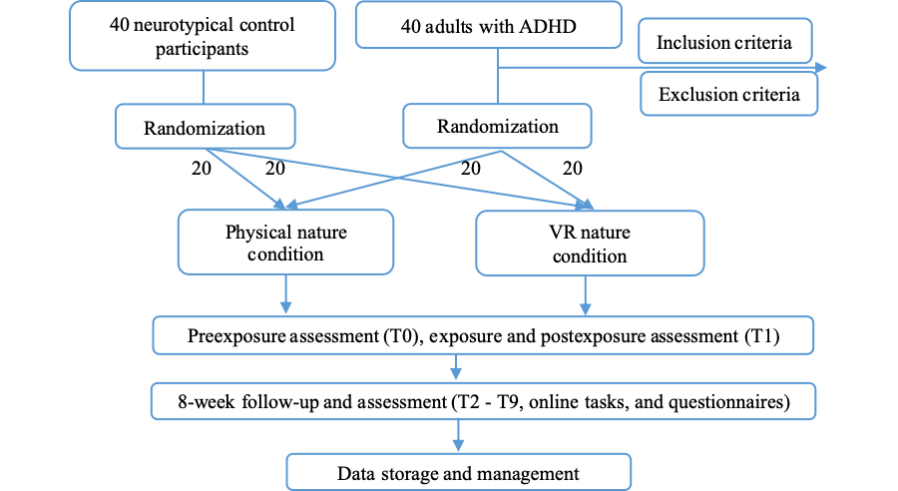
Participant flow and schedule of assessments.

As illustrated in Figure 1, cognitive and emotional assessments will be administered at 10 time points: preexposure (T0), immediately postexposure (T1), and weekly over an 8-week follow-up period (T2-T9). These assessments will be completed online within the study platform. ADHD-specific assessments will be conducted at 3 time points: T0, T1, and the final follow-up (T9). EEG data will be recorded continuously during the exposure session to capture real-time brain activity in response to the environmental condition.

The initial baseline assessment (T0) will take approximately 30 to 40 minutes, while T1 and T9 will require approximately 20 to 30 minutes because of the inclusion of ADHD symptom self-reports. The weekly follow-up assessments from T2 to T8 will each take approximately 10 to 15 minutes to complete. T0, T1, and T9 assessments will be conducted at the study site, where trained research assistants will guide participants through the procedures using their smartphones. All follow-up assessments (T2-T8) will be completed independently by participants at home via the online platform. To support retention during the 8-week follow-up, participants will receive automated weekly reminders via the Neureka app, with 1 additional email reminder if a survey is missed. Small nonmonetary incentives (eg, feedback summary upon study completion) will be offered to encourage continued participation.

### Follow-Up Assessments

Existing work on nature exposure largely falls into 2 patterns: single-session studies that examine immediate effects and multiweek intervention programs involving repeated exposure (eg, weekly 45-minute walks in nature), with follow-up data suggesting that some benefits are no longer detectable at 1-month follow-up [64]. Against this background, this study includes an extended follow-up after a single 20-minute exposure to real or VR-simulated nature, delivered via an online survey platform that sends automatic weekly reminders before each assessment to support retention and minimize participant burden.

Beyond the immediate postexposure assessment, participants complete weekly online follow-ups over an 8-week period. The primary rationale for these repeated assessments is to characterize how any acute changes in cognition and mood observed at T1 decay over time. This design allows us to estimate the short-term time course of attenuation and to explore whether the pattern of change differs between exposure modalities and between individuals with different neurocognitive profiles, including varying levels of ADHD symptoms. Such information may help to inform the scheduling and dosing of future nature-based interventions.

In addition, the extended 8-week window provides an opportunity to examine more gradual shifts in lifestyle and nature relatedness. At baseline and at follow-up, we record the frequency and duration of participants’ additional contact with real outdoor nature and VR-based nature experiences, as well as repeated measures of nature connectedness using the Nature Connection Index (NCI) [65]. These data will be used descriptively and in exploratory analyses to assess whether a single experimental session has any downstream impact on everyday nature exposure or perceived connection to nature while also helping to account for ongoing environmental input when interpreting long-term trajectories.

### Participants

A total of 80 participants aged 18 to 65 years will be recruited, comprising 40 adults with a confirmed diagnosis of ADHD and 40 neurotypical controls.

Adults with ADHD will be recruited through outpatient ADHD clinics in Sligo, Ireland, where psychologists or psychiatrists will identify eligible participants based on a clinical diagnosis according to *DSM-5* criteria. Neurotypical controls will be recruited through community outreach, including advertisements in schools and local public spaces.

Inclusion criteria for all participants include fluency in English and the ability to understand study instructions and complete cognitive assessments. Exclusion criteria for all participants include (1) a history of learning disabilities; (2) severe brain injury causing cognitive impairment; (3) current substance or alcohol abuse; (4) severe medical illnesses or mental illnesses like acute psychosis, mania, or major depression affecting cognitive function; and (5) extended reality–specific contraindications, including a history of epilepsy or photosensitive seizures, diagnosed vestibular disorders (eg, Ménière disease, chronic vertigo, and vestibular migraine), or severe motion sickness or cybersickness that has previously limited engagement with VR or similar technologies. These factors may confound cognitive and EEG outcomes, limiting the interpretability of environmental exposure effects, and are therefore excluded to ensure participant safety. For the neurotypical control group, participants will be excluded if they score above the clinical threshold (≥15) on the General Health Questionnaire-12 (GHQ-12) [66], or if they meet the ADHD screening criteria on the Adult ADHD Self-Report Scale (ASRS) Part A (4 or more responses fall within the darkly shaded boxes, indicating symptoms highly consistent with adult ADHD) [67].

### Exposure Conditions

#### Physical Nature Exposure Condition

Participants assigned to this condition will be escorted to a designated seating area within a forest located near the clinic. They will be instructed to sit quietly for 20 minutes, remain awake, and keep their eyes open. The study will be conducted between April and September, when local weather and daylight conditions in the west of Ireland are relatively stable. Sessions will be scheduled on dry, calm days and, where possible, at comparable times of day to minimize variation in temperature and light.

For each session, the research team will record ambient temperature, weather conditions (eg, sun, cloud cover, wind, or unexpected rain), and notable environmental events (eg, unusual noise or human activity). These contextual variables will be retained for sensitivity and exploratory analyses and will be described as inherent features of the real nature condition. Immediately following the exposure, participants will complete a series of cognitive and emotional assessments outdoors in the same natural setting.

#### Immersive VR Exposure Condition

Participants assigned to this condition will be equipped with the Meta Quest 3 standalone headset and hand controllers. They will engage in a 20-minute immersive simulation of a forest environment, designed to closely approximate the real forest setting used in the physical nature condition. The simulation is delivered through an open-source forest scene compatible with the Meta Quest platform. The VR application is a nonclinical research stimulus and is used solely for experimental exposure in this study. It includes synchronized visual and auditory stimuli (eg, trees and ambient forest sounds such as bird calls) to enhance ecological realism. Immediately following the VR exposure, participants will complete the same assessments as those in the physical nature condition.

#### Exposure Duration

Both exposure conditions use a fixed 20-minute session. This duration was selected to balance efficacy and tolerability, particularly for adults with ADHD. From a safety perspective, a review of temporal aspects of simulator sickness indicates that longer VR exposure is one of the strongest predictors of cybersickness, with more severe symptoms often emerging after approximately 20 to 30 minutes of continuous use [68]. In parallel, work on VR applications for individuals with attentional difficulties suggests that prolonged continuous tasks can lead to vigilance decrement, frustration, and disengagement in people with ADHD [69]. Keeping the exposure at 20 minutes therefore provides a conservative upper bound that aims to minimize the risk of sensory overload and cybersickness while maintaining engagement, especially in the group with ADHD.

At the same time, 20 minutes is sufficient to capture meaningful restorative effects. Although dose-response data for EEG in natural environments are limited, field studies of nature exposure in daily life have shown that approximately 20 minutes in urban green spaces can already produce measurable changes in stress-related biomarkers and autonomic regulation [70]. Given that EEG indexes neural activity on a millisecond timescale, a 20-minute window is expected to provide ample data to observe changes in cognitive processing and brain dynamics during exposure without extending into durations where fatigue and simulator sickness become more common. Taken together, a 20-minute session was judged to offer an adequate “dose” to elicit and measure neural and cognitive responses to real and VR-simulated nature while reducing the likelihood of adverse effects and dropout, particularly among adults with ADHD.

#### Safety and Tolerability Monitoring

All VR sessions will be conducted individually in a quiet room with a researcher present at all times. Before donning the headset, participants will be informed that they may stop the session at any point if they experience discomfort (eg, nausea, dizziness, eye strain, or anxiety) without providing a reason and without any negative consequences. The researcher will visually monitor posture and behavior throughout the 20-minute exposure and will terminate the session immediately if marked distress or instability is observed.

Immediately after VR exposure, participants will complete the Virtual Reality Sickness Questionnaire (VRSQ) [71] to systematically assess cybersickness symptoms. Any adverse events or early terminations will be documented using a standard adverse event form, and participants will be offered rest and, if needed, medical review according to site procedures. Participants who request to stop the session will not be asked to complete any further study procedures at that visit, and no additional outcome data will be collected beyond what has already been obtained.

### Data Collection and Instruments

#### Overview

This study will integrate cognitive assessments, ecological momentary assessments (EMA), standardized questionnaires, and EEG recordings to evaluate cognitive performance, emotional well-being, and neural activity across both exposure conditions. Demographic data (gender, age, and occupation) and medication taken at the day of assessment will be self-reported. Cognitive and emotional data will be collected using the nonprofit Neureka app [72], developed by the Trinity College Dublin Gillan Laboratory. The app incorporates mobile technology and citizen science approaches to monitor well-being and cognition in real-world contexts. It will be used to administer baseline and follow-up assessments over an 8-week monitoring period. All data collected through Neureka will be stored and processed following the European Union General Data Protection Regulation (GDPR). Additional questionnaires, including the GHQ-12, ASRS, and other standardized clinical measures of ADHD, will be administered on paper during in-person assessments.

#### Demographic and Contextual Measures

Demographic information (age, gender, education, and medication status) will be collected via a brief self-report questionnaire at baseline. The same form will also assess participants’ contact with natural environments over the previous 2 weeks, including the frequency and approximate duration of visits to outdoor green and blue spaces and any use of VR-based nature applications. These contextual variables will be used to characterize the sample and explore whether recent nature exposure moderates acute and follow-up outcomes.

#### Cognitive Assessments

Meta Mind: a gamified metacognitive task in which participants make confidence judgments after completing a series of perceptual decisions involving dot quantity discrimination [73].

Star Racer: a gamified adaptation of the Trail Making Test [74], designed to measure cognitive flexibility and executive control through alternating visuospatial tracking and task-switching components.

#### Emotional and Psychological Measures

EMA surveys containing 17 items adapted from the Quick Inventory of Depressive Symptomatology will track real-time fluctuations in mood, anxiety, and subjective cognitive well-being over the 8 weeks.

GHQ-12 will be administered to neurotypical controls as a screening tool for psychological distress. Participants scoring above the clinical threshold (a Likert score of 15 or higher) will be excluded.

The 6-item NCI will be administered at baseline and at the final follow-up to assess participants’ perceived connection with the natural world. Scores will be used to examine whether a single exposure to real or VR-simulated nature alters nature connectedness and whether baseline NCI moderates cognitive and emotional responses.

#### ADHD-Related Measures

To assess ADHD symptomatology and its functional impact, the following standardized instruments will be used:

ASRS: a widely used self-report questionnaire assessing the severity of ADHD symptoms across inattention and hyperactivity/impulsivity dimensions. All participants will complete the ASRS to enable dimensional modeling across the full sample.

Adult ADHD Quality of Life Questionnaire (AAQoL): a 29-item self-report scale measuring the impact of ADHD on quality of life across multiple domains [75]. This measure will be administered to participants in the group with ADHD.

ADHD Clinical Outcome Self-Report (ACOS-Self): a 15-item routine clinical outcome measure assessing ADHD symptoms, associated mental health challenges, and functional impairments [76]. It will also be used only with participants formally diagnosed with ADHD.

#### VR Tolerability Measures

The VRSQ will be completed immediately after the VR exposure. The VRSQ provides a brief assessment of oculomotor and disorientation symptoms related to cybersickness. In addition, participants in the VR condition will rate (1) how strongly they felt “as if really being in the forest” and (2) the overall comfort and tolerability of the VR experience on 7-point Likert scales. Together, these measures will be used to monitor participant safety, document tolerability and perceived presence of the VR condition, and, in exploratory analyses, examine whether cybersickness or low tolerability are associated with cognitive or EEG outcomes.

#### EEG Recording and Preprocessing

Neural activity will be recorded during the exposure session using a mobile 32-channel system (EMOTIV FLEX 2 Gel), which combines a research-grade amplifier with a wireless gel-based cap optimized for naturalistic and VR settings. The montage includes 32 active scalp sites (AF7, AF3, AF4, Fp1, Fp2, F1, Fz, F2, F3, F4, FC1, FCz, FC2, C1, Cz, C2, CP1, CPz, CP2, P1, Pz, P2, P7, P8, PO7, PO8, PO3, POz, PO4, O1, Oz, and O2), plus a Common Mode Sense and Driven Right Leg reference pair placed at the linked earlobes, providing high-impedance noise reduction and compatibility with standard ADHD biomarkers such as frontal-midline theta, P3, and theta/beta ratio [77-82]. Signals will be sampled at 500 Hz with a direct current to 45 Hz hardware passband. Participants’ scalps will be cleaned with alcohol wipes, and conductive gel will be applied until electrode impedance is ≤5 kΩ (absolute ceiling, 10 kΩ) with left-right symmetry within 1 kΩ for homologous pairs, to ensure stable signal-to-noise for both spectral and event-related potential (ERP) measures. Cables will be secured along the cap with Velcro, and the amplifier will be fixed at the occipital strap to minimize movement artifacts during seated exposure and tasks. This mobile configuration has been widely used in naturalistic and VR research to capture reliable spectral power and ERP components while allowing participants to remain in ecologically valid environments and enables comparable acquisition in both the outdoor forest and headset-based VR conditions.

Resting-state baselines will be collected preexposure and postexposure (T0 and T1) with 2-minute eyes-open (fixation cross) and 2-minute eyes-closed recordings while participants sit quietly, instructed to stay relaxed, minimize blinking, and avoid large movements. During the 20-minute nature or VR exposure, EEG will be recorded continuously while participants remain seated; they will be reminded that they may pause or terminate the session if they experience discomfort, and any early termination will be documented and treated as dropout for EEG analysis. Evidence from previous VR-EEG studies [83] and from our own pilot testing indicates that simultaneous use of the FLEX cap and the VR headset is safe and allows reliable signal acquisition. Nevertheless, because VR head-mounted display padding can occasionally impinge on frontal or frontopolar sites, headset fitting will follow a predefined substitution protocol: if AF3/AF4 are partially obstructed, frontal alpha asymmetry will be computed from the canonical F3/F4 pair; if Fp1/Fp2 cannot be used, priority will be given to maintaining midline integrity at Fz. For any substituted electrodes, 3D electrode positions will be digitized to allow offline correction of spatial shifts, and the midline chain Fz-Cz-Pz-Oz will be preserved in all participants for core spectral and ERP analyses.

Raw data will be exported from the proprietary EMOTIV format and converted to European Data Format/BioSemi Data Format for analysis. Offline preprocessing will be performed in EEGLAB and FieldTrip, using a 0.1 to 45 Hz finite-impulse-response band-pass filter and a 50 Hz notch filter to remove mains interference while preserving upper-beta activity relevant for theta/beta ratio outcomes. Data will then be re-referenced to the average of all scalp channels. Continuous recordings will be visually inspected to mark gross artifacts (eg, cable pops or sudden movement) and sections with poor contact. Independent component analysis will be applied to identify and remove stereotyped ocular and muscle components, following established guidelines for mobile and VR EEG. Remaining data will be segmented into 2-second nonoverlapping epochs for resting-state spectral analyses and into −200 to 800 ms epochs time locked to task stimuli or responses for ERP measures (N2/error-related negativity, P3, and related indices). Epochs showing extreme amplitudes or improbable distributions (as indexed by EEGLAB joint-probability and kurtosis metrics) will be rejected, ensuring that only artefact-free data contribute to power and ERP estimates.

To address concerns about VR-specific noise, exposure sessions will be conducted with participants seated, with head and body motion restricted to comfortable postural adjustments. Headset straps will be adjusted to avoid direct pressure on electrodes, and any pressure-related noise will be monitored online via impedance checks and offline via topographical inspection. Because the same EEG hardware, montage, and preprocessing pipeline are used in both exposure conditions, any residual artifacts linked to the outdoor environment (eg, occasional muscle adjustments due to temperature) or to the VR headset will be treated symmetrically and accounted for at the analysis stage (eg, sensitivity analyses excluding highly contaminated channels or sessions). This rigorous acquisition and preprocessing protocol is designed to ensure that observed changes in theta/beta ratio, frontal midline theta, posterior alpha power, and ERP components can be interpreted as meaningful indices of cognitive engagement and restoration rather than artifacts of the recording setup.

#### Data Management and Confidentiality

All study data will be pseudonymized using unique participant IDs before analysis. Identifiable information (eg, consent forms and contact details for follow-up reminders) will be stored separately from research data on encrypted, password-protected institutional servers with access restricted to the core research team. Neureka app data will be stored on secure servers hosted by Trinity College Dublin and exported only in deidentified form. In line with GDPR and institutional policies, deidentified research data will be retained for 10 years and then securely destroyed. Study findings will be communicated in aggregate form to participating clinical teams and, on request, to individual participants via a plain-language summary. Results will also be disseminated through peer-reviewed publications, conference presentations, and an update to the trial registry record once outcomes are available.

Given the minimal-risk, single-session behavioral nature of this study, no independent data monitoring committee will be established; study conduct and protocol adherence will instead be overseen by the principal investigator and study team in line with institutional and ethics-committee requirements. No formal interim analyses or statistical stopping rules are planned, but the principal investigator and the research ethics committee retain the authority to pause or terminate the study early should any unanticipated safety concerns arise.

### Statistical Analysis

#### Outcomes

The primary outcomes of this study will be: (1) changes in indices of neural restoration and engagement (parietal-occipital alpha power, frontal midline theta, and frontal alpha asymmetry) from preexposure to postexposure; (2) performance on two cognitive tasks: Meta Mind (confidence score, accuracy, and reaction time) to evaluate metacognition, and Star Racer (completion time, error rate, and task-switching cost) to measure cognitive flexibility and executive control; and (3) ADHD symptom severity, assessed by ASRS, included in both diagnostic group comparisons and dimensional modeling across the full sample.

Secondary outcomes will include exploratory trajectories of emotional well-being assessed through EMA, as well as self-reported nature connectedness (NCI) and weekly real-life contact with natural environments. These repeated measures are intended to characterize short-term decay or change patterns after a single exposure. Clinical change in the group with ADHD will be measured with the ACOS-Self, and quality of life with the AAQoL. Measures of VR tolerability and cybersickness (VRSQ) will be analyzed descriptively and, where appropriate, as covariates to aid interpretation of cognitive and EEG outcomes.

#### Power

Sample size estimation focused on the primary mechanistic outcomes of the study, namely preexposure to postexposure changes in EEG alpha power and performance on the 2 cognitive tasks as a function of diagnostic group (ADHD vs neurotypical) and exposure condition (real nature vs VR nature). Power calculations were conducted in G*Power (version 3.1; Heinrich Heine University Düsseldorf), using a 2 × 2 × 2 mixed-model ANOVA (group × condition × time: preexposure vs postexposure). Pilot data from a comparable ADHD sample [84] indicated a pre-to-post change in alpha amplitude of 6.93 μV (SD 2.04) at baseline and 7.88 μV (SD 1.83) postintervention, corresponding to a Cohen *d* of 0.49 (moderate effect size) for the within-subject contrast. Assuming a correlation of 0.5 among repeated measures, this maps onto a repeated-measures ANOVA effect size in the moderate range.

Using a 2-tailed α of .05 and desired power of 0.80, the required total sample size was calculated to be 64 to 72 participants (approximately 16 to 18 per cell in the 2 × 2 design) to detect effects in this range on the primary EEG outcome. To accommodate potential attrition and data loss (eg, unusable EEG recordings), a 20% buffer was applied, resulting in a final recruitment target of 80 participants (n=20 per subgroup). This sample size is expected to provide adequate statistical power to detect moderate pre-post effects and group × condition interactions on alpha power and core cognitive outcomes.

Secondary and exploratory analyses (eg, EMA trajectories, dimensional ASRS scores, and follow-up data) will be modeled using linear mixed-effects approaches in the same sample. These models are intended to characterize patterns of change over time and symptom-related gradients in responsiveness; findings from these analyses will therefore be interpreted with appropriate attention to effect sizes and precision estimates rather than relying solely on hypothesis-testing thresholds. The planned sample provides ≥80% power to detect moderate pre-post changes in alpha power for the primary group × condition × time effect in the planned linear mixed-effects analysis. Analyses of higher-order interactions and secondary outcomes (eg, EMA trajectories) will be considered exploratory and interpreted cautiously.

#### Statistical Methods

All analyses will follow an intention-to-treat approach and will be conducted using R (R Foundation for Statistical Computing). Statistical tests will be 2-tailed with a significance level of α=.05. Descriptive statistics will summarize sample characteristics. Baseline group differences will be assessed using Pearson chi-square tests for categorical variables and independent-samples *t* tests (or nonparametric equivalents) for continuous variables. Any variables showing systematic baseline imbalance will be considered as covariates in sensitivity analyses.

To evaluate the primary outcomes—changes from preexposure (T0) to immediate postexposure (T1) in EEG alpha power, cognitive performance (Meta Mind and Star Racer), and ASRS, we will implement linear mixed-effects models (LMMs) using the *lme4* package. Fixed effects will include exposure condition (real vs VR nature), diagnostic group (ADHD vs neurotypical), time (T0 vs T1, with T9 added where relevant to examine short-term maintenance), and their interactions. Participant will be entered as a random intercept; random slopes for time will be added where supported by convergence and model fit. The primary contrasts of interest are: (1) group × condition × time for alpha power in predefined frontal and parietal regions of interest, and (2) condition × time and group × time interactions for cognitive and ASRS outcomes.

For EEG data, log-transformed alpha power (8-13 Hz) will be averaged within each region of interest and exposure phase. Within-subject change scores (T1-T0, and T9-T0 where applicable) will be analyzed in the LMM framework described above. Exploratory time-resolved effects across the 20-minute exposure will be examined using cluster-based permutation tests on epoch-wise alpha power, with family-wise error controlled at 0.05. Effect sizes will be reported as Cohen *d* for pairwise contrasts and partial eta squared (η^2^_p_) for omnibus tests.

Secondary outcomes include trajectories of emotional well-being assessed via weekly EMA, clinical functioning in the group with ADHD (ACOS-Self), quality of life (AAQoL), and nature connectedness (NCI). EMA scores over T0-T9 will be modeled with LMMs including fixed effects for time (treated as a continuous or categorical variable depending on fit), condition, group, and their interactions, with random intercepts for participants. ACOS-Self, AAQoL, and NCI change from T0 to T9 will be analyzed using separate LMMs with fixed effects for group, condition, and their interaction. Self-reported real-world nature and VR exposure during follow-up will be explored as time-varying covariates in additional models to probe their contribution to decay or maintenance of effects.

To incorporate a dimensional perspective, *z*-scored ASRS total and subscale scores will be included as continuous predictors in a restricted set of prespecified models [85] (eg, ASRS × condition × time interactions for alpha power and cognitive outcomes). These analyses will test whether attentional trait variation predicts sensitivity to environmental exposure across the full sample, without overparameterizing models.

Multiple-comparison control will be applied at the level of outcome families: EEG (regions of interest and key contrasts), cognitive tasks (Meta Mind and Star Racer indices), EMA trajectories, and clinical measures. Within each family, *P* values will be adjusted using false discovery rate procedures; unadjusted and adjusted values will both be reported.

Model assumptions (normality of residuals, homoscedasticity, and, where relevant, sphericity) will be checked using diagnostic plots and formal tests. If assumptions are violated, transformations or robust alternatives will be considered. Missing data will primarily be handled within the mixed-model framework under a missing-at-random assumption. In addition, sensitivity analyses will be conducted using multiple imputation and complete-case subsets (participants with data at least up to T1 and T9) to examine the impact of attrition on key inferences.

## Results

The project received funding in May 2025. Participant recruitment is anticipated to begin in January 2026, followed by data collection starting in April 2026 to ensure suitable outdoor environmental conditions. Given the minimal-risk, single-session nature of the intervention, no independent data monitoring committee has been established; safety is overseen by the principal investigator, and all adverse events are recorded and reviewed after each session. Data analysis will proceed thereafter, and the study results are expected to be published in May 2028.

## Discussion

This study addresses a key gap in understanding how nature, both physical and virtual, interacts with distinct neurocognitive profiles to influence cognition, well-being, and brain activity. By combining behavioral tasks, self-report and symptom measures, and mobile EEG, it is designed to test whether VR-simulated nature can reproduce the short-term cognitive and neural changes associated with real-world natural exposure and whether it does so via similar or different processes. Rather than treating VR as a straightforward substitute, the protocol explicitly compares patterns of attentional engagement, alpha-based indices of restoration, and symptom-related variability across the 2 exposure types.

Focusing on adults with ADHD is intended to add both scientific and clinical value. Scientifically, it allows examination of how environmental stimulation interacts with atypical attentional and regulatory systems, using both diagnostic group comparisons and dimensional modeling of ADHD symptom scores. Clinically, it explores whether a single, low-intensity nature-based session, real or virtual, may support attentional regulation and quality of life in a population often underserved by traditional therapeutic approaches. Including both ADHD and neurotypical participants and analyzing symptom dimensions across the full sample should help distinguish effects that are broadly shared from those that are amplified or attenuated in ADHD.

The protocol also extends current work on nature exposure by combining immediate postexposure assessments with an 8-week follow-up. Weekly online measures of mood, stress, cognitive complaints, and nature connectedness (including self-reported contact with real and VR nature) will allow us to explore how any initial benefits change over time and whether decay patterns differ by exposure condition or symptom profile. These longitudinal data are exploratory rather than confirmatory but may provide useful information for designing the frequency and “dose” of future nature-based interventions.

In parallel, the integration of mobile EEG in both real and VR settings is intended to provide converging neurophysiological evidence on attentional engagement restoration. Alpha power and related spectral measures will be examined alongside behavioral and self-report outcomes, with the aim of identifying neural signatures that correspond to subjective restoration or cognitive effort. Although the EMOTIV FLEX system offers only moderate spatial resolution compared with laboratory-based EEG, its portability enables ecologically valid recording during naturalistic exposure, which is central to the aims of this protocol.

Several limitations should be acknowledged. The study compares real nature with VR-simulated nature but does not include a nonnature digital control condition, which limits inferences about whether observed effects are specific to natural content vs immersion or digital engagement more broadly. Individual differences in sensory sensitivity and simulator sickness may affect responses to the VR condition despite screening, safety monitoring, and the use of a relatively short (20-minute) exposure. EEG collected during movement-constrained outdoor and VR sessions will still be susceptible to muscle and motion artifacts, and results will depend on rigorous preprocessing and conservative quality control. Conducting the real-nature condition outdoors inevitably introduces variability in weather, temperature, light, ambient noise, and unexpected stimuli. To limit extreme discrepancies, data collection will be scheduled within months of relatively stable weather, sessions will be arranged flexibly to avoid adverse conditions, and key environmental characteristics (eg, temperature, wind, noise level, and light intensity) will be logged to support transparent reporting and exploratory adjustment. In addition, although dimensional modeling improves ecological and theoretical validity, estimates of interaction effects may be constrained by the planned sample size and by measurement noise in self-report data.

Despite these constraints, the study is expected to make a cautious but meaningful contribution to environmental neuroscience, digital health, and neurodiversity research. By directly comparing real and VR nature in adults with and without ADHD, and by tracking both short-term changes and their subsequent attenuation, the protocol aims to clarify when VR can approximate the benefits of real nature and when modality or cognitive profile matters. The findings may help to refine hypotheses about the mechanisms of nature-related restoration and to inform pragmatic decisions about when immersive VR is a suitable, acceptable, and safe option for expanding access to nature-based experiences.

## Appendix

Multimedia Appendix 1 SPIRIT (v2025) checklist.

## Abbreviations

AAQoL: Adult ADHD Quality of Life Questionnaire
ACOS-Self: ADHD Clinical Outcome Self-Report
ADHD: attention-deficit/hyperactivity disorder
ANZCTR: Australian New Zealand Clinical Trials Registry
ART: Attention Restoration Theory
ASRS: Adult ADHD Self-Report Scale
DSM-5: Diagnostic and Statistical Manual of Mental Disorders, Fifth Edition
EEG: electroencephalography
EMA: ecological momentary assessments
ERP: event-related potential
fMRI: functional magnetic resonance imaging
GDPR: General Data Protection Regulation
GHQ-12: General Health Questionnaire-12
LMM: linear mixed-effects model
NCI: Nature Connection Index
SRT: Stress Reduction Theory
VR: virtual reality
VRSQ: Virtual Reality Sickness Questionnaire
